# Construction of A Preliminary Three-Dimensional Structure *Simian betaretrovirus* Serotype-2 (SRV-2) Reverse Transcriptase Isolated from Indonesian Cynomolgus Monkey

**DOI:** 10.21315/tlsr2020.31.3.4

**Published:** 2020-10-15

**Authors:** Uus Saepuloh, Diah Iskandriati, Joko Pamungkas, Dedy Duryadi Solihin, Sela Septima Mariya, Dondin Sajuthi

**Affiliations:** 1 Primate Research Centre, Bogor Agricultural University (PSSP LPPM IPB), Jalan Lodaya II/5 Bogor 16151, Indonesia; 2 Department of Biology, Faculty of Mathematics and Natural Sciences, Bogor Agricultural University, Kampus Darmaga, Bogor 16680, Indonesia; 3 Faculty of Veterinary Medicine, Bogor Agricultural University, Kampus Darmaga, Bogor 16680, Indonesia

**Keywords:** Reverse Transcriptase, SRV-2 Indonesian Isolates, 3D Structure Model

## Abstract

*Simian betaretrovirus* serotype-2 (SRV-2) is an important pathogenic agent in Asian macaques. It is a potential confounding variable in biomedical research. SRV-2 also provides a valuable viral model compared to other retroviruses which can be used for understanding many aspects of retroviral-host interactions and immunosuppression, infection mechanism, retroviral structure, antiretroviral and vaccine development. In this study, we isolated the gene encoding reverse transcriptase enzyme (RT) of SRV-2 that infected Indonesian cynomolgus monkey (Mf ET1006) and predicted the three dimensional structure model using the iterative threading assembly refinement (I-TASSER) computational programme. This SRV-2 RT Mf ET1006 consisted of 547 amino acids at nucleotide position 3284–4925 of whole genome SRV-2. The polymerase active site located in the finger/palm subdomain characterised by three conserved catalytic aspartates (Asp90, Asp165, Asp166), and has a highly conserved Y*M*DD motif as Tyr163, Met164, Asp165 and Asp166. We estimated that this SRV-2 RT Mf ET1006 structure has the accuracy of template modelling score (TM-score 0.90 ± 0.06) and root mean square deviation (RMSD) 4.7 ± 3.1Å, indicating that this model can be trusted and the accuracy can be seen from the appearance of protein folding in tertiary structure. The superpositionings between SRV-2 RT Mf ET1006 and Human Immunodeficiency Virus-1 (HIV-1) RT were performed to predict the structural in details and to optimise the best fits for illustrations. This SRV-2 RT Mf ET1006 structure model has the highest homology to HIV-1 RT (2B6A.pdb) with estimated accuracy at TM-score 0.911, RMSD 1.85 Å, and coverage of 0.953. This preliminary study of SRV-2 RT Mf ET1006 structure modelling is intriguing and provide some information to explore the molecular characteristic and biochemical mechanism of this enzyme.

**Highlights**

- Gene encoding reverse transcriptase enzyme of *Simian betaretrovirus serotype-2* (RT SRV-2) infected Indonesian long-tailed monkey was isolated and molecular characterised.
- We predicted the 3D structure model of this RT SRV-2 enzyme using computational program.
- This 3D model provides domains function, enzymatic site active and hybrid DNA/ RNA-enzyme interaction.

## INTRODUCTION

Retroviral reverse transcriptase (RT) is a multifunctional enzyme that catalyses the formation of a double-stranded DNA from the single stranded retroviral RNA genome. This complex process is called reverse transcription, a critical step in the life cycle of all retroviruses which is responsible for viral genome replication. The newly synthesized DNA is then integrated into the host genome by integrase, another retroviral enzyme (Telesnitsky & Goff 1997). The retroviral RT enzyme has multiple function, such as an RNA-dependent DNA polymerase, a DNA dependent DNA polymerase, DNA-directed RNA cleavage, strand transfer, and strand displacement synthesis (Herschhorn & Hizi 2010). The RT enzyme has two enzymatic activities, as a DNA polymerase and RNase H activities localised in two separate protein domains. The DNA polymerisation function is capable of using either DNA or RNA as a template and RNase H function serves to hydrolyse the RNA strand within an RNA/DNA hybrid. Both the polymerase and RNase H activities are essential for viral replication (Coté & Roth 2008; Telesnitsky & Goff 1997).

RTs from the different groups of retroviruses share similar functional catalytic activities but they significantly differ in particular parameters, such as structure and subunit composition, molecular weights, catalytic properties, biochemical and biophysical characteristics and sensitivity to different inhibitors (Herschhorn & Hizi 2010). The RT of type-1 Human Immunodeficiency Virus (HIV-1) is the most intensively studied enzymes in the last decades (Herschhorn & Hizi 2010). The HIV-1 RT is a major target for the development of antiretroviral drug therapy of HIV-1, the virus causing acquired immunodeficiency syndrome (AIDS), with over half of current FDA-approved therapeutics against HIV infection targeting this enzyme (Ilina *et al*. 2012; Sarafianos *et al*. 2009). As a major target for anti-HIV therapy, RT has been the subject of extensive research using crystal structure determinations and biochemical assays (Sarafianos *et al*. 2009). HIV-1 RT has been characterised structurally. The structures of complexes with dsDNA (Ding *et al*. 1998); dsDNA and the incoming nucleotide (Huang *et al*. 1998); and an RNA/DNA hybrid (Sarafianos *et al*. 2001) are available.

*Simian betaretrovirus* serotype-2 (SRV-2) is a causative agent of simian acquired immunodeficiency syndrome in Asian macaques it accounts for high morbidity and mortality in research and breeding facilities (Marx *et al*. 1984; Gardner *et al*. 1988; Lerche 2010; Montiel 2010; Lerche & Osborn 2003). The SRV-2 genome contains four genes organised in the order 5′-*gag-prt-pol-env*-3′ and encode identically in size of 8105-bp proviruses (Marracci *et al*. 1995; Marracci *et al*. 1999). Similar to other retroviruses, SRV-2 can transcribe its own RNA genome into a double-stranded DNA by the action of a reverse transcriptase enzyme. The resulted double-stranded DNA is inserted randomly into the chromosome of the host cell and can express the genetic information of the retrovirus (Marracci *et al*. 1999). The presence of gene encoding RT enzyme in SRV-2 genome may potentially be studied and utilised further as RT enzyme model for developing an anti RT drug of other retroviruses especially HIV/AIDS.

In this study, we have amplified the gene encoding the RT enzyme of the SRV-2 isolated from an infected Indonesian cynomolgus monkey and predicted the three dimensional (3D) structure model of this SRV-2 RT enzyme using I-TASSER (Iterative Threading ASSEmbly Refinement) computational program. I-TASSER is a computational method that has been successful in accurately modelling protein structures (Zhang 2008; Roy *et al*. 2010; Yang *et al*. 2015). I-TASSER uses a combinatorial approach, employing all three conventional methods for structure modelling: comparative modelling, threading, and *ab initio* modelling (Roy *et al*. 2010).

## MATERIALS AND METHODS

### PCR Amplification and Sequencing of SRV-2 Reverse Transcriptase Gene

The DNA from archived peripheral blood mononuclear cells (PBMCs) of Indonesian cynomolgus monkey (the ID was ET1006) that positive to SRV-2 was extracted using QiaAmp DNA blood minikit (Qiagen, Hilden, Germany). PCR amplification of 2.5 μL DNA was carried out with 2U P*fx* DNA polymerase (Thermo Fisher Scientific, Carlsbad, USA), in 25 μL total reaction containing 2 mM MgCl_2_, 1x PCR buffer, 1 mM dNTPs (Thermo Fisher Scientific, Carlsbad, USA). We developed the specific primer using Primer3 program (Untergasser *et al*. 2012) based on SRV-2 complete genome (AF126467.1) as SRV-2 RT 3284F (5′-CCTGTTTGGGTTGATC-3′) and SRV-2 RT 4925R (5′-GTGATTACCTTGAGATAAAGGTCC-3′). PCR amplification was performed for 35 cycles in the following condition: denaturation at 94°C for 30 s, annealing at 55°C for 30 s, and extention at 68°C for 1.5 min. Amplicon (12.5 μL) were visualised on 1.0% TAE agarose gel and analysed with GelDoc machine (BioRad, California, USA). PCR product was gel purified using Qiaquick gel extraction kit (Qiagen, Hilden, Germany) then sequenced in First Base Laboratories Sdn Bhd (Malaysia). The primer used for sequencing was similar to that used for PCR amplification and some internal specific primers: SRV-2 RT 3751R (5′-GCCCAAGATTTTCTGATTGG-3′), SRV-2 RT 3799F (5′-GATTGGCGAA CAAGTTTTGC-3′), SRV-2 RT 4247F (5′-TACTGGCCTCTTCTGG -3′), and SRV-2 RT 4604F (5′-GGCATAGCCGCATACACTTT-3′).

### Phylogenetic Tree Analysis

The obtained nucleotide sequences were analysed using BioEdit program (Hall 1999) and alignment was performed with computer software BLAST program (Altschul *et al*. 1997) (https://blast.ncbi.nlm.nih.gov/Blast.cgi). Nucleotide encoded amino acid translation was performed using EMBOSS Transeq Program (Madeira *et al*. 2019) (http://www.ebi.ac.uk/Tools/emboss/transeq). Amino acid sequences were aligned using the ClustalW2 (Madeira *et al*. 2019) (https://www.ebi.ac.uk/Tools/msa/clustalw2/) then adjusted to phylogenetic tree construction using MEGA 6.0 program based on Neighbour-Joining method (Tamura *et al*. 2013). Genetic distances were estimated using Kimura’s two parameter method (Kimura 1980) and the bootstrap analysis was performed 500 replicates to asign confidence to tree nodes. RT amino acid sequences of others retroviruses were used as references: Avian Myeloblastosis Virus (AMV, AAB31929.1), Baboon Endogenous Virus (BaEV, BAA89659.1), Feline Leukemia Virus (FeLV, BAL04139.1), Feline Immunodeficiency Virus (FIV, ABO69501.1), Human Immunodeficiency Virus-1 (HIV-1, ADN28092.1), HIV-2 (AAB01352.1), Moloney Murine Leukemia Virus (MMLV, NP_955591.1), Mason-Pfizer Monkey Virus (MPMV, AAA47711.1), Simian Foamy Virus (SPF, YP_001961122.1), Simian Endogenous Retrovirus (SERV, AAC97565.1), Simian Immunodeficiency Virus (SIV, AAA74707.1), Simian Retrovirus-1 (SRV-1, AAA47732.1), SRV-2 (AAD43256.1), SRV-2 (AAA47562.1), SRV-4 (YP_003864102.1), SRV-5 (BAM71050), SRV-8 (AOS48113.1), Human T-Lymphotropic Virus-2 (HTLV-2, AAB59885.1), Simian T-Lymphotropic Virus-1 (STLV-1NP_049560.1) and Mouse Mammary Tumour Virus (MMTV, BAA03767).

### Functional Structure and Three Dimensional Model of SRV-2 RT Enzyme

Amino acid sequences were aligned using the ClustalW2 and the secondary-structure predictions were made based on sequences and structural motif using I-TASSER (https://zhanglab.ccmb.med.umich.edu/I-TASSER/). The conserve domain and amino acids position of active site were refined manually based on predicted secondary structure and multiple sequences alignment as previously defined of HIV-1 (1HYS A) (Sarafianos *et al*. 2001) and SRV-2 *pol* AAD43256 (Marracci *et al*. 1999). The I-TASSER program was used to determine the function of the query protein inferred by structurally matching the predicted 3D models against the proteins of known structure and function in the Protein Data Bank (PDB). The functional analogs from the global search results are ranked based on the conserved structural patterns present in the model, measured using a scoring scheme that combines template modelling (TM-score), root-mean-squared deviation (RMSD), sequence identity, and coverage of the structure alignment. TM-score was defined to assess the topological similarity of protein structure pairs with a value in the range of (0, 1), a higher score indicating better structural match. The results from the global and local search are combined to present a comprehensive list of functional analogs (Zhang 2008; Roy *et al*. 2010). Matching structures were analysed using PyMol program (Delano 2002) (http://www.pymol.org) that allowed us to further evaluate possible structural and functional domains of the SRV-2 RT protein.

## RESULTS AND DISCUSSIONS

### PCR Amplification and Sequencing of SRV-2 Reverse Transcriptase Gene

**Figure 1:**
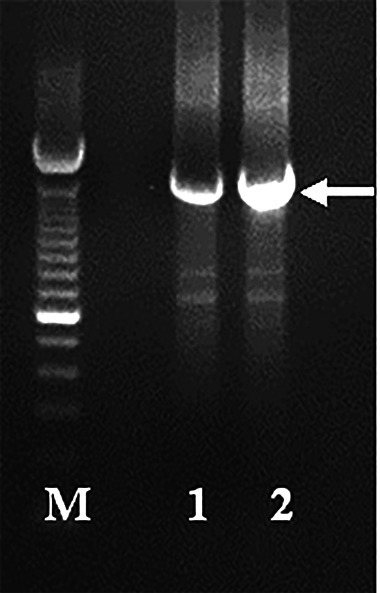
PCR amplification to SRV-2 RT gene isolated from Indonesian cynomolgus monkeys (SRV-2 RT Mf ET1006) resulting 1641 bp amplicon. (M) 100 bp DNA ladder (Invitrogen), (1) SRV-2 RT Mf ET1006, (2) SRV-2/A549 positive control (obtained from National Primate Research Centre, University of Washington, USA).

Recently, we have isolated and characterised the SRV-2 from wild Indonesian cynomolgus monkeys with sequences analysis result of this virus envelope region revealed high homology (97%–98% similarities) to SRV-2 reported previously in captive macaques in US Primate Research Centre (Iskandriati *et al*. 2010). In this research, we analysed the RT encoding gene from Indonesian SRV-2 (SRV-2 RT Mf ET1006) since there has been no report focusing on SRV-2 RT study. Therefore, this preliminary research may provide a better understanding of structure and mechanism of retroviral SRV-2 RT. The DNA amplification to SRV-2 RT Mf ET1006 resulted in 1641 bp amplicon (Fig. 1) at the position of 3284–4925 of whole provirus SRV-2 genome consisting of 547 amino acids as enzymatic RT. These SRV-2 RT Mf ET1006 amino acids have 98%–99% similarities to others SRV-2 previously isolated from captive macaques in some primate research centre in US (AF126467, AF126468, and M16605) and have 79%–87% similarities to others SRVs (SRV 1, 3, 4, 5, 8 and SERV) (Table 1). It seemed that the amino acid sequences of RT enzyme among SRVs are corserved.

### Phylogenetic Tree Analysis of SRV-2 RT

**Figure 2:**
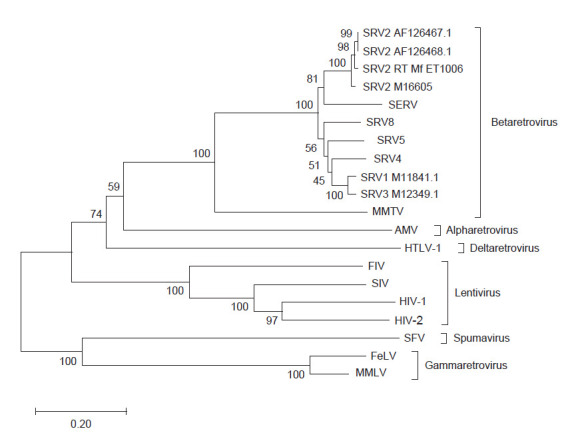
Phylogenetic tree of SRV-2 RT Mf ET1006 amino acids sequences relatedness to others retroviruses constructed using the neighbor-joining method. The bootstrap percentages of 500 replicate are shown at the branch nodes. The evolutionary distances are estimated by branch lengths of the tree indicated and drawn to scale, which represents the number of base substitutions per site.

We have multiple-aligned our amino acid sequences and constructed phylogenetic tree to determine genetic relationship among RT enzyme family retroviruses (Fig. 2). The phylogenetic result showed that the target sequences of SRV-2 RT Indonesian isolate (SRV-2 RT Mf ET1006) have the closes relationship with SRV-2 D2/RHE/OR (AF126467 and AF126468) first isolated from rhesus monkey at Oregon Regional Primate Research Centre US in 1999 (Marracci *et al*. 1999). SRV-2 RT Mf ET1006 was clustered with endogenous SERV, SRV-1, SRV-3, SRV-4, SRV-5, SRV-8 and MMTV as *betaretrovirus* subfamily. This subfamily has the closest relationship with *alpharetrovirus* (AMV), then both of this cluster branched with the *deltaretrovirus* subfamily (HTLV-1). *Alpha*, *beta* and *deltaretrovirus* subfamily then branched with *lentivirus* where HIV-1 as a member of this subfamily is the most investigated retrovirus in biomedical field. The furthest relation of SRV cluster was *spumavirus* and *gammaretrovirus* where the MMLV as a member of this subfamily has widely applied RT enzyme in RT-PCR technique. This data has the similar correlation with previously reported research by Li *et al*. (1995) on *pol* retrovirus phylogenetic from 55 retroelements where SRV-1 is closely related to MPMV (SRV-3) and evolved after SRV-2. Therefore, the highly conserved *pol* gene region has been the target in phylogenetic studies both in nucleotide and amino acids sequences (Li *et al*. 1995).

### Functional Structure Prediction of SRV-2 RT Enzyme

**Figure 3:**
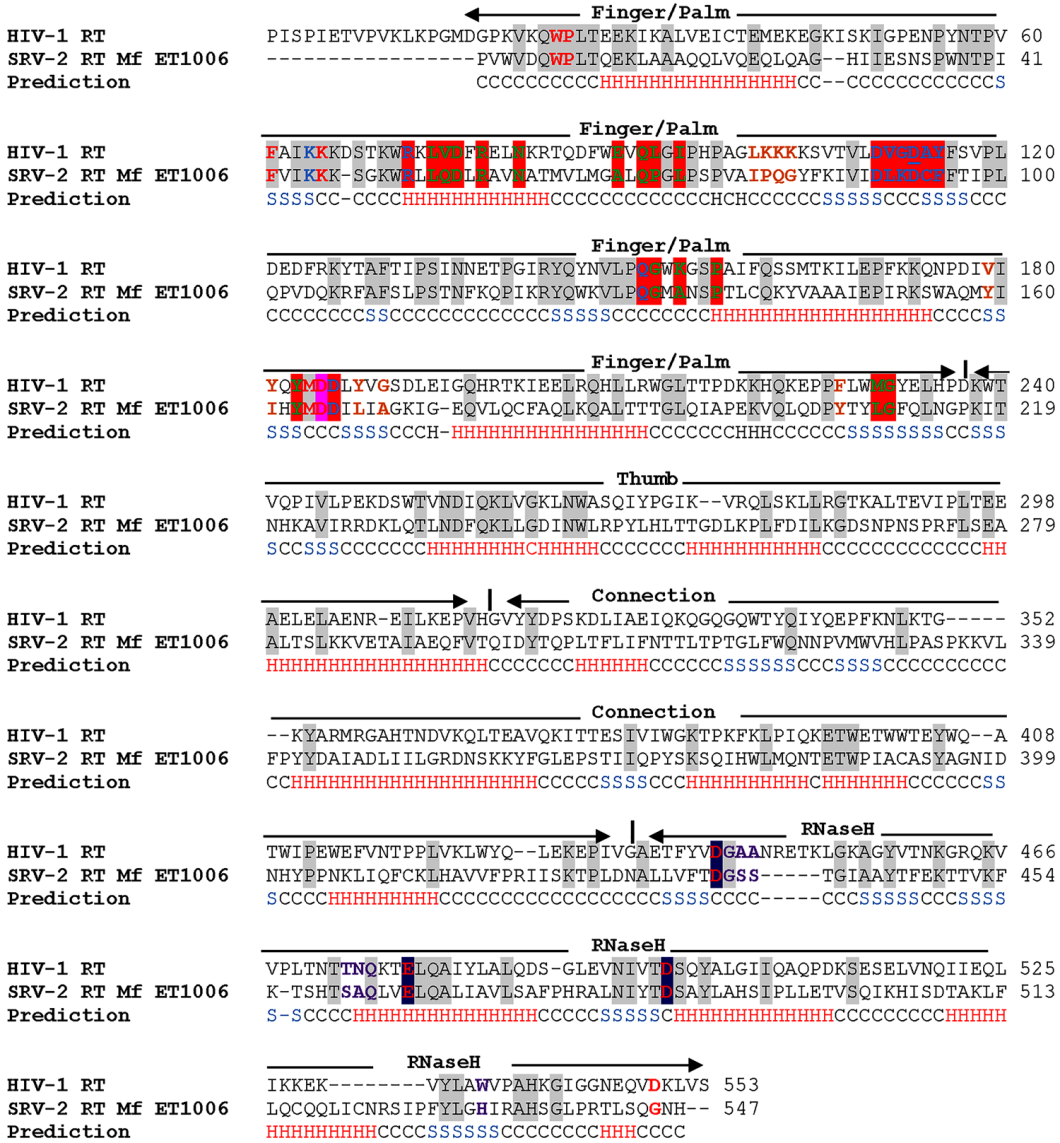
Amino acid sequences alignment and secondary structural motifs prediction of the SRV-2 RT Mf ET1006 and HIV-1 RT. Amino acid sequence of SRV-2 RT Mf ET1006 from the position 1–547 and HIV-1 RT from 18–553 were aligned based on sequences and structural motifs using the programs ClustalW, STRAP2 and I-TASSER. The motifs were indicated as α-helixes (H), β-sheets (S), and coils (C). The amino acids active site was indicated in red and highlighted red, the DNA binding sequences were indicated in green, dNTPs binding site were indicated in blue, NNRTI binding site were indicated in brown, RNA/DNA hybrid (RNase H) were indicated in purple and highlighted purple. Meanwhile, the Y*X*DD sequences were over-scored with a red line. The boundaries of the Finger/Palm, Thumb, Connection and RNase H were shown with a vertical line.

In this study, we tried to compare the functional structure of SRV-2 RT Mf ET1006 with HIV-1 RT. The comparison was based on the HIV-1 RT crystal structure in PDB (2B6A and 1HYS) and SRV-2 *pol* previously reported in GenBank (AAD43256.1). Amino acid sequences of SRV-2 RT Mf ET1006 at the position of 1–547 and HIV-1 RT from 1–553 were aligned using ClustalW, STRAP2 (Gille & Frömmel 2001) and I-TASSER (Fig. 3). Comparison between HIV-1 RT p66 andSRV-2 RT Mf ET1006 showed that finger/palm subdomain has 32% similarity of 237 residues, the thumb subdomain has 25.6% similarity of 82 residues, the connection subdomain has 12% similarity of 133 residues, and the RNase H subdomain has 27% similarity of 118 residues. Overall, the full-length comparison between SRV-2 RT Mf ET1006 and HIV-1 RT has 25.5% of 553 residues. Meanwhile, if the very conservative amino acid (such as Ala vs. Val, Leu vs. Ile, etc.) was allowed for comparison, it resulted in 43% similarities.

The polymerase active site that is located in the finger/palm subdomain is characterised by three conserved catalytic aspartates at the position of Asp90, Asp165, Asp166 for SRV-2 RT Mf ET1006, and Asp110, Asp185, Asp186 for HIV-1 RT (Table 2). This triad aspartates (the last two residues are part of Y*X*DD motif), play a significant role in the HIV-1 RT enzymatic catalytic through the binding to the divalent cation Mg^2+^ (Sharma *et al*. 2005). This leads to the assumption that the catalytic active site of SRV-2 RT Mf ET1006 coordinate with two divalent ions, Mg^2+^. The Y*X*DD motif, where X is a variable amino acid, is highly conserved and plays a critical role among various viral RNA dependent DNA polymerases. The conserved motif of SRV-2 RT Mf ET1006 and HIV-1 RT consists of four amino acids, YMDD for Tyr, Met, Asp, Asp at the position 163–166 for SRV-2 RT Mf ET1006 and 183–186 for HIV-1 RT. SRV-2 RT Mf ET1006 has a YMDD amino acid motif similar with HIV-1, SIV and FIV, however, it was different from YVDD motif that MMLV and FeLV have (Sharma *et al*. 2005). Recently, many studies focused on this Y*X*DD motif due to its significance in NRTI resistance, RT fidelity, polymerase process, and viral replication (Sharma *et al*. 2005). The dNTPs binding sites is also located in the polymerase active site and have conserve residues which are Lys64, Arg72, Asp110, Asp113, Gln151, and Asp186 for HIV-1 RT. Meanwhile Lys45, Arg52, Asp90, Asp93, Gln131, and Asp166 for SRV-2 RT Mf ET1006. The RNase H active sites that interacted with DNA/RNA hybrid contain conserved DED motif such as Asp, Glu, and Asp at the position of 443, 478, and 498 for RNase H of HIV-1 RT and 436, 465, and 486 for RNase H of SRV-2 RT (Table 2 and Fig. 3).

### Three Dimensional Structure Model of SRV-2 RT Mf ET1006

**Figure 4:**
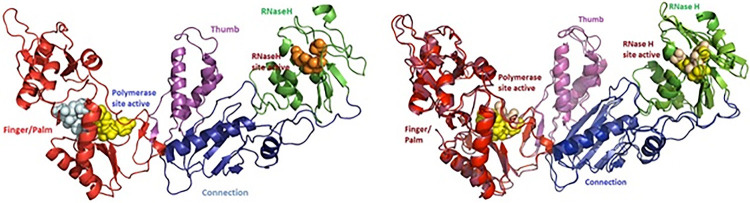
(A) Three-dimensional structure model of the full-length SRV-2 RT Mf ET1006. The models were generated using I-TASSER method and visualised using Pymol program. The estimated accuracy of model is 0.90 ± 0.06 (TM-score), 4.7 ± 3.1Å (RMSD) and C-score = 1.33. The fingers/palm, thumb, connection and RNaseH subdomains are coloured in dark red, purple, dark blue and green, respectively. Light blue spheres represent the residues of the polymerase active site and dNTPs binding site. Yellow spheres represent the Y*X*DD motif in the finger/palm. Meanwhile, the brown spheres indicated the DED motif of RNaseH active site. (B) Superpositioning model between SRV-2 RT Mf ET1006. with HIV-1 RT (2B6A.pdb), estimated accuracy is 0.911 (TM-score), 1.85 Å (RMSD), and 0.953 (coverage).

Three-dimensional structure prediction of the full-length SRV-2 RT Mf ET1006 is depicted in Fig. 4(A). The models were generated using I-TASSER method and visualised using Pymol program. Five models of SRV-2 RT Mf ET1006 were computationally generated based on pair-wise structure similarity and the Model I structure gave a highest C-score at 1.33 compared to Models 2 to 5 with C-score less than −0.1. The C-score is a confidence score which ranges from −5 to 2, with higher scores representing higher confidence in the model. The C-score has a strong correlation with the quality of the I-TASSER models (Roy *et al*. 2010). In general, models with C-score > −1.5 are expected to have a correct fold. The RMSD and TM-score are both well-known measures of topological similarity between the model and native structure. TM-score values range from 0 to 1, where a higher score indicates a better structure match (Roy *et al*. 2010). The model I structure of SRV-1 RT Mf ET1006 has the estimated accuracy at TM-score 0.90 ± 0.06 with RMSD 4.7 ± 3.1Å. Therefore, the Model I SRV-2 RT Mf ET1006 structure has the best C-score. So, it can be trusted, and the accuracy can be seen from the appearance of protein folding in tertiary structure (Fig. 4). The superpositionings between HIV-1 RT and SRV-2 RT Mf ET1006 were performed to predict the structural details and to optimise best fits for illustrations. Table 2, Fig. 3 and Fig. 4(B) showed the structural motifs used in the superposition comparisons between SRV-2 RT Mf ET1006 and HIV-1 RT. The HIV-1 RT structures possessing the most consistent and well-defined motifs for our study were 1HYS (Sarafianos *et al*. 2001), 2HMI (Ding *et al*. 1998) and 2B6A (Roth *et al*. 1997). This SRV-2 RT Mf ET1006 structure model has the highest homology to HIV-1 RT (2B6A) with estimated accuracy at TM-score 0.911, RMSD 1.85 and coverage of 0.953 measured with TM-align from Zhang Lab (Zhang & Skolnick 2005).

**Figure 5:**
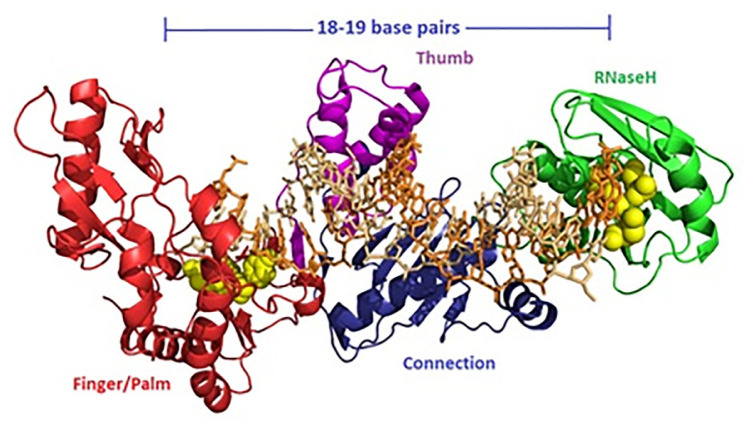
Ribbon diagram of the SRV-2 RT Mf ET1006 bound to a stick model of an 18–19-base RNA/DNA duplex oligonucleotide substrate. The various domains of the enzyme were colour-coded as presented in Fig. 4. The active site amino acids of the DNA polymerase and RNase H are shown in yellow spheres.

The models of the SRV-2 RT Mf ET1006 in complex with 18/19-mer DNA/RNA nucleic acid substrates stretching from the polymerase active site to the RNase H active site are depicted in Fig. 5. The SRV-2 RT Mf ET1006 was superpositioning to HIV-1 RT (1N6Q) (Sarafianos *et al*. 2002) based on I-TASSER template proteins with similar binding site. The assessment of this model was carried out using TM-align server (http://zhanglab.ccmb.med.umich.edu/TM-align) with TM-score 0.838, Identity 0.258 and BS-score 1.48. BS-score is a measure of local similarity (sequence and structure) between template binding site and predicted binding site in the query structure. Based on large scale benchmarking analysis, BS-score > 1.1 reflects a very good local match between the predicted and template binding site (Roy *et al*. 2010).

**Figure 6:**
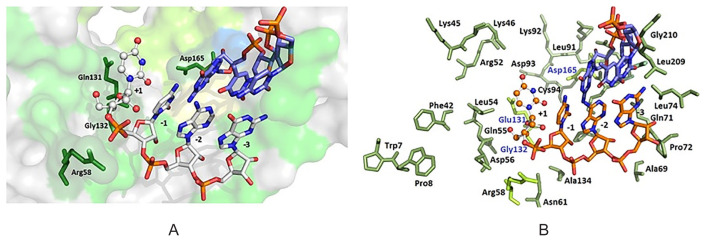
Interaction of amino acids that play roles in polymerase site active of SRV-2 RT Mf ET1006 with terminal portion of the RNA/DNA duplex. DNA was indicated with red and blue stick, while RNA in red, white and blue stick, signed with −1, −2 and −3 as duplex RNA/ DNA; overhang RNA was indicated by +1 as round edges stick. (A) SRV-2 RT Mf ET1006 site active is represented in green surface with conserved amino acids Asp165, Gln131, Gly132 and Arg58 (shown in green stick); (B) SRV-2 RT Mf ET1006 amino acids site active represented in green stick.

The model of interaction between SRV-2 RT Mf ET1006 site actives with nucleic acids was developed based on the available crystal structure HIV-1 RT (Huang *et al*. 1998) (Fig. 6). The amino acids involved in interaction with incoming dNTP of HIV-1 RT such as Lys65, Arg72, Asp113, Ala114, Tyr115 and Gln151 (Huang *et al*. 1998), have a conserved amino acid with SRV-2 RT Mf ET1006 as Lys45, Arg52, Asp93, Gln131. Ala114 and Tyr115 in HIV-1 RT were replaced with Cys94 and Phe95, in SRV-2 RT Mf ET1006. The substitution of Tyr115 with Phe caused a little deleterious effect in enzyme catalytic activities that interact directly with deoxyribose of the incoming triphosphate (Boyer *et al*. 2000). We predicted the first nucleotide overhang of the template that will pair with incoming dNTP and interact with side chain of Leu54 and against the backbone of Gly132 (Figs. 6(A) and (B)). The second and third 5′-template overhang will pack against the residues of Trp7, Pro8 and Phe42. Direct interaction of bases in minor groove occur with Pro137, Met164, Leu74 and Tyr163. The incoming dNTP pairs with the templating bases in which the triphosphate residues will interact with Lys45, Arg52, Asp93 and Cys94. Meanwhile, the 3′-OH of dNTP will line by side chain of Asp93, Phe95, Gln131 and the peptide backbone between 93 and 95.

In this study, a predicted three-dimensional structure of SRV-2 RT Mf ET1006 enzyme should be very informative and useful for understanding its structure and functional characteristics, since no structure is currently available for this enzyme. This is because the structure of a protein provides precise molecular details that often facilitate experimental characterisation of an expected function. The development and validation of computational methods that can predict protein structure to a relatively high level of accuracy and thereby facilitate functional annotation, biochemical analyses, and biological characterisation are a high priority. Our model provides an initial structural framework for understanding the SRV-2 RT Mf ET1006 domains function, enzymatic site active and hybrid DNA/ RNA-enzyme interaction.

## CONCLUSION

The preliminary study of three-dimensional structure modelling of monomeric SRV-2 RT Mf ET1006 compared to heterodimeric HIV-1 RT is intriguing, given some information about the similarities and differences of amino acid sequences between them to predict the same function of each subdomains. Using the structural model studies to explore the molecular characteristic and biochemical mechanism of SRV-2 RT Mf ET1006 and to understand the mechanism of enzymatic catalytic would provide valuable information. Recently, many RT structures have been explored and reported in PDB Data Bank. Therefore, these available RT structures have contributed and inspired us to explore the SRV-2 RT Mf ET1006 structure based on three-dimensional structure models. This will intrigue us to expand our knowledge about SRV-2 RT Mf ET1006 as a virus model of HIV and develop new improved therapeutics agents.

## Figures and Tables

**Table 1:** Amino acid identities of SRV-2 RT Mf ET1006 compared with others SRVs.

		1	2	3	4	5	6	7	8	9	10
1	SRV2 RT Mf ET1006	–	99	99	98	86	86	83	82	84	82
2	SRV2 AF126467		–	99	97	85	85	83	82	83	81
3	SRV2 AF126468			–	97	85	85	83	82	83	81
4	SRV2 M16605				–	85	85	82	81	84	82
5	SRV1					–	96	85	84	86	79
6	SRV3						–	85	84	85	79
7	SRV4							–	82	83	77
8	SRV5								–	81	77
9	SRV8									–	79
10	SERV										–

**Table 2:** Amino acid comparison of selected structural motifs of SRV-2 RT Mf ET1006 compared to HIV-1 RT.

Domain/subdomain motif	SRV-2 RT Mf ET1006 amino acid^a^	HIV-1 RT amino acid^b^
Finger/palm	1–216	1–237
Thumb	217–297	238–316
Connection	298–430	317–437
RNase H	431–547	438–553
Polymerase active site	Trp7, Pro8, Phe42, Lys46, Arg52, Leu54, Gln55, Asp56, Arg58, Asn61, Ala69, Gln71, Pro72, Leu74, Asp90, Leu91, Lys92, Asp93, Cys94, Phe95, Gln131, Gly132, Ala134, Pro137, Tyr163, Asp165, Asp166, Leu209, Gly210	Trp24, Pro25, Phe61, Lys65, Arg72, Leu74, Val75, Asp76, Arg78, Asn81, Glu89, Gln91, Leu92, Ile94, Asp110, Val111, Gly112, Asp113, Ala114, Tyr115, Gln151, Gly152, Lys154, Pro157, Tyr183, Asp185, Asp186, Met230, Gly231
RNA/DNA hybrid	Ser438, Ser439, Thr440, Ala461, Ala488, His531, Arg533, Ala534	Ala446, Arg448, Glu449, Thr473, Asn474, Gln475, Gln500, Hi539
RNase active site	Asp436, Glu465, Asp486	Asp443, Glu478, Asp498
dNTPs binding site	Lys45, Arg52, Asp90, Leu91, Lys92, Asp93, Cys94, Phe95, Gln131, Asp166	Lys64, Arg72, Asp110, Val111, Gly112, Asp113, Ala114, Tyr115, Gln151, Asp186
YXDD	Tyr163, Met164, Asp165, Asp166	Tyr183, Met184, Asp185, Asp186

*Notes*:

a SRV-2 RT Mf ET1006 was aligned based on I-TASSER result and referred to SRV-2 *pol* (GenBank AAD43256.1)

b HIV-1 RT based on PDB of 2B6A,1HYS and referred to HIV-1 pol (GenBank ADN28092.1)

## References

[b1-tlsr-31-3-47] Altschul SF, Madden TL, Schaffer AA, Zhang J, Zhang Z, Miller W, Lipman DJ (1997). Gapped BLAST and PSI-BLAST: A new generation of protein database search programs. Nucleic Acids Research.

[b2-tlsr-31-3-47] Boyer PL, Sarafianos SG, Arnold E, Hughes SH (2000). Analysis of mutations at position 115 and 116 in the dNTPs binding site of HIV-1 reverse transcriptase. PNAS USA.

[b3-tlsr-31-3-47] Coté ML, Roth MJ (2008). Murine leukemia virus reverse transcriptase: Structural comparison with HIV-1 reverse transcriptase. Virus Research.

[b4-tlsr-31-3-47] Delano WL (2002). PyMOL: An open-source molecular graphics tool. CCP4 Newsletter On Protein Crystallography.

[b5-tlsr-31-3-47] Ding J, Das K, Hsiou Y, Sarafianos SG, Clark AD, Jacobo-Molina A, Tantillo C, Hughes SH, Arnold E (1998). Structure and functional implications of the polymerase active site region in a complex of HIV-1 RT with a double-stranded DNA template-primer and an antibody Fab fragment at 2.8 A resolution. Journal of Molecular Biology.

[b6-tlsr-31-3-47] Gardner MB, Luciw P, Lerche N, Marx P (1988). Nonhuman primate retrovirus isolates and AIDS. Advances Veterinary Sciences Comparative Medicine.

[b7-tlsr-31-3-47] Gille C, Frömmel C (2001). STRAP: Editor for STRuctural Alignments of Proteins. Bioinformatics.

[b8-tlsr-31-3-47] Hall TA (1999). BioEdit: A user-friendly biological sequence alignment editor and analysis program for Windows 95/98/NT. Nucleic Acids SymposiumSeries.

[b9-tlsr-31-3-47] Herschhorn A, Hizi A (2010). Retroviral reverse transcriptase. Cell Molecular Life Science.

[b10-tlsr-31-3-47] Huang H, Chopra R, Verdine GL, Harrison SC (1998). Structure of a covalently trapped catalytic complex of HIV-1 reverse transcriptase: Implications for drug resistance. Science.

[b11-tlsr-31-3-47] Ilina T, LaBarge K, Sarafianos SG, Ishima R, Parniak MA (2012). Inhibitors of HIV-1 reverse transcriptase-associated Ribonuclease H activity. Biology.

[b12-tlsr-31-3-47] Iskandriati D, Saepuloh U, Mariya S, Grant RF, Solihin DD, Sajuthi D, Pamungkas J (2010). Isolation and characterization of *simian retrovirus* type D from *Macaca fascicularis* and *M. nemestrina* in Indonesia. Microbiologi Indonesia.

[b13-tlsr-31-3-47] Kimura M (1980). A simple method for estimating evolutionary rates of base substitutions through comparative studies of nucleotide sequences. Journal of Molecular Evolution.

[b14-tlsr-31-3-47] Lerche NW, Osborn KG (2003). *Simian retrovirus* infections: Potential confounding variables in primate toxicology studies. Toxicology Pathology.

[b15-tlsr-31-3-47] Lerche NW (2010). *Simian retroviruses*: Infection and disease-implications for immunotoxicology research in primates. Journal of Immunotoxicology.

[b16-tlsr-31-3-47] Li MD, Bronson DL, Lemke TD, Faras AJ (1995). Phylogenetic analysis of 55 retroelements on the basis of the nucleotide and product amino acid sequences of *pol* gene. Molecular Biology Evolution.

[b17-tlsr-31-3-47] Madeira F, Park YM, Lee J, Buso N, Gur T, Madhusoodanan N, Basutkar P, Tivey ARN, Potter SC, Finn RD, Lopez R (2019). The EMBL-EBI search and sequence analysis tools APIs in 2019. Nucleic Acids Research.

[b18-tlsr-31-3-47] Marracci GH, Kelley RD, Pilcher KY, Crabtree L, Shiigi SM, Avery N, Leo G, Webb MC, Hallick LM, Axthelm MK (1995). Simian AIDS type D serogroup 2 retrovirus: Isolation of an infectious molecular clone and sequence analyses of its envelope glycoprotein gene and 3′ long terminal repeat. Journal of Virology.

[b19-tlsr-31-3-47] Marracci GH, Avery NA, Shiigi SM, Couch G, Palmer H, Pilcher KY, Nichols H, Hallick LM, Axthelm MK, Machida CA (1999). Molecular cloning and cell-specific growth characterization of polymorphic variants of type D serogroup 2 simian retroviruses. Virology.

[b20-tlsr-31-3-47] Marx PA, Maul DH, Osborne KG (1984). Simian AIDS: Isolation of type D retrovirus and disease transmission. Science.

[b21-tlsr-31-3-47] Montiel NA (2010). An updated review of *simian betaretrovirus* (SRV) in macaque hosts. Journal of Medical Primatology.

[b22-tlsr-31-3-47] Roth T, Morningstar ML, Boyer PL, Hughes SH, Buckheit RW, Michejda CJ (1997). Synthesis and biological activity of novel non-nucleoside inhibitors of HIV-1 reverse transcriptase 2-Aryl-substituted benzimidazoles. Journal of Medical Chemistry.

[b23-tlsr-31-3-47] Roy A, Kucukural A, Zhang Y (2010). I-TASSER: A unified platform for automated protein structure and function prediction. Nature Protocol.

[b24-tlsr-31-3-47] Sarafianos SG, Das K, Tantillo C, Arthur D, Clark J, Jianping D, Whitcomb JM, Boyer PL, Hughes SH, Arnold E (2001). Crystal structure of HIV-1 reverse transcriptase in complex with a polypurine tract RNA: DNA. EMBO Journal.

[b25-tlsr-31-3-47] Sarafianos SG, Clark AD, Das K, Tuske S, Birktoft JJ, Ilankumaran P, Ramesha AR (2002). Structures of HIV-1 reverse transcriptase with pre- and post-translocation AZTMP-terminated DNA. EMBO Journal.

[b26-tlsr-31-3-47] Sarafianos SG, Marchand B, Das K, Himmel D, Parniak MA, Hughes SH, Arnold E (2009). Structure and function of HIV-1 reverse transcriptase: Molecular mechanisms of polymerization and inhibition. Journal of Molecular Biology.

[b27-tlsr-31-3-47] Sharma PL, Nurpeisov V, Schinazi RF (2005). Retrovirus reverse transcriptase containing a modified YXDD motif. Antiviral Chemical Chemotherapy.

[b28-tlsr-31-3-47] Tamura K, Stecher G, Peterson D, Filipski A, Kumar S (2013). MEGA6: Molecular evolutionary genetics analysis version 6.0. Molecular Biology and Evolution.

[b29-tlsr-31-3-47] Telesnitsky A, Goff SP, Coffin JM, Hughes SH, Varmus HE (1997). Reverse transcriptase and the generation of retroviral DNA. Retroviruses.

[b30-tlsr-31-3-47] Untergasser A, Cutcutache I, Koressaar T, Ye J, Faircloth BC, Remm M, Rozen SG (2012). Primer3-new capabilities and interfaces. Nucleic Acids Research.

[b31-tlsr-31-3-47] Yang J, Yan R, Roy A, Xu D, Poisson J, Zhang Y (2015). The I-TASSER suite: Protein structure and function prediction. Nature Methods.

[b32-tlsr-31-3-47] Zhang Y, Skolnick J (2005). TM-alignment: A protein structure alignment algorithm based on the TM-score. Nucleic Acid Research.

[b33-tlsr-31-3-47] Zhang Y (2008). I-TASSER server for protein 3D structure prediction. BMC Bioinformatics.

